# Atteinte cardiaque au cours de la dystrophie myotonique de Steinert: expérience marocaine, à propos de 18 cas

**DOI:** 10.11604/pamj.2015.20.131.2751

**Published:** 2015-02-16

**Authors:** Ghita Saghi, Rachida Bouhouch, Loubna Salaheddine, Nezha Birouk, Salama Nadifi, Ibtissam Fellat, Mohamed Cherti

**Affiliations:** 1 Service de Cardiologie B, Maternité Souissi, CHU Ibn Sina, Rabat, Maroc; 2 Service de Neurophysiologie, Hôpital des Spécialités, CHU Ibn Sina, Rabat, Maroc; 3 Service de Neurogénétique, CHU Ibn Rochd, Casablanca, Maroc

**Keywords:** Dystrophie myotonique de Steinert, atteinte cardiaque, cœur, Steinert myotonic dystrophy, Cardiac involvement, heart

## Abstract

La maladie de Steinert ou dystrophie myotonique de type 1 (DM1) est une maladie génétique à transmission autosomique dominante caractérisée par une myotonie et une atteinte de plusieurs organes dont le cœur. L'atteinte cardiaque est la plus grave des atteintes systémiques puisqu'elle conditionne le pronostic vital. Ce travail a pour but de déterminer les anomalies cardiaques rencontrées au cours de la DM1 et de mettre en exergue l'intérêt d'un examen cardiaque rigoureux et régulier, indépendamment de la sévérité de l'atteinte neuromusculaire, ainsi que l'apport des examens cardiaques complémentaires et notamment l'exploration électrophysiologique. 18 patients atteints de DM1 ont bénéficiés d'une exploration cardiaque systématique. Il s'agit de 9 hommes et de 9 femmes, d’âge moyen de 41,8 +/- 16,2 ans. 66 p.100 des patients sont symptomatiques sur le plan cardiovasculaire. Les anomalies électrocardiographiques sont dominées par un trouble de la conduction intra-ventriculaire dans 16 p.100 des cas et un BAV de 1er degré dans 16 p.100 des cas. L'Holter ECG objective une hyperexcitabilité à l’étage atrial et/ou ventriculaire dans 50p.100 des cas. L'ETT est normale chez 95 p.100 des patients. L'exploration électrophysiologique, réalisée chez 4 patients symptomatiques, a objectivé un bloc tronculaire dans un cas ayant conduit à l'implantation d'un PM double chambre. Un seul patient est décédé suite à une détresse respiratoire. Enfin, on n'a pas noté de corrélation entre l'atteinte cardiaque et neuromusculaire. Une exploration cardiaque est indispensable chez tout patient atteint de DM1, en dépit de l'absence de symptômes, et un bilan annuel minimal s'impose pour guetter un éventuel trouble rythmique et/ou conductif, fatal en l'absence de traitement adéquat.

## Introduction

La maladie de Steinert ou DM1 est une maladie génétique à transmission autosomique dominante caractérisée par une myotonie (lenteur à la décontraction musculaire) et une atteinte multisystémique: musculaire, neurologique, oculaire, respiratoire, digestive, endocrinienne et cardiaque. Il s'agit de la plus fréquente des dystrophies musculaires héréditaires de l'adulte avec une prévalence de 1 à 10 cas pour 100 000 habitants [1]. Elle se caractérise sur le plan génétique par une mutation du gène codant pour la protéine DMPK (myotonic dystrophy protein kinase) situé sur le bras long du chromosome 19 (19q13, 3) [2] et une expansion du triplet cytosine-thymine-guanine (CTG) (plus de 50 triplets), avec comme corollaire une accumulation intranucléaire de l'ARN complémentaire des CTG et réduction du taux de la protéine DMPK. Cette dernière est ubiquitaire mais s'exprime essentiellement dans le muscle squelettique et cardiaque. L'atteinte cardiaque est fréquente et, bien que souvent asymptomatique, conditionne le pronostic vital et explique en partie le taux élevé de mort subite rencontré dans cette affection.

## Méthodes

18 patients atteints de dystrophie myotonique de type 1 (DM1) et appartenant à 9 familles ont été colligés au service de cardiologie B (laboratoire de Rythmologie) sur une période de 3 ans s’écoulant de septembre 2009 à décembre 2012. Ces patients ont été vus initialement au service de Neurologie où le diagnostic de DM1 a été retenu en se basant sur l'histoire clinique, la myotonie, les données de l’électromyogramme et l’étude génétique. Les patients ont bénéficiés d'un examen ophtalmologique, endocrinien et digestif puis ils ont été adressés dans notre structure à la recherche systématique d'une atteinte cardiaque associée. L'exploration cardiaque comporte un examen clinique, un électrocardiogramme, une échocardiographie transthoracique, un Holter rythmique de 24 heures, et pour quelques patients on a eu recours à une épreuve d'effort, un ECG haute amplification, une coronarographie et une étude électrophysiologique (EEP).

## Résultats

Notre série comprend 9 hommes et 9 femmes. Leur âge moyen est de 41,8 +/- 16,2 ans (âges extrêmes: 14 à 71ans) et l’âge moyen au moment du diagnostic est de 38,2 +/- 14,3 ans (âges extrêmes: 14 à 69 ans). La durée d’évolution de la maladie est de 4 ans en moyenne.

### Atteinte neurologique

Le motif de consultation initial est une fatigabilité à la marche. L'examen neurologique objective une myotonie chez tous les patients ainsi qu'un déficit musculaire distal et trouble de la marche à type de steppage dans 44% des cas. Par ailleurs, on retrouve un ptosis dans 55% des cas, une parésie faciale dans 72% des cas et un trouble de la déglutition dans 22% des cas. L’électromyogramme réalisé chez tous nos patients est de type myogène, et l’étude de l'acide-désoxyribo-nucléique (ADN) leucocytaire a confirmé la dystrophie myotonique de Steinert en objectivant une expansion de triplets CTG supérieure à 37 répétitions. Aucun des patients n'est grabataire. En outre, on note un trouble de la vigilance et de la fonction cognitive chez deux patients.

### Atteinte cardiaque

12 patients (soit 66% des cas) présentent des signes fonctionnels cardiovasculaires avec une prédominance de dyspnée et de lipothymie (Tableau 1). Aucun patient n'est en insuffisance cardiaque. L'ECG de base objective des troubles de la conduction chez 6 patients: un BAV 1^er^ degré dans 3cas(P12, P14, P17) et un trouble de conduction intracardiaque à type de BBDI et BBGC dans 3 cas (P3, P9, P1) (Tableau 2). Seuls 50% de ces patients sont symptomatiques (Tableau 3). Par ailleurs, on note une bradycardie sinusaleasymptomatiquechez un patient (P10), une onde p micro-voltée chez une patiente (P8) aux antécédents de syncope; et un syndrome de Wolf Parkinson White (WPW) dans un cas (P4). L'Holter ECG, réalisé systématiquement chez tous nos patients, objectivedes anomalies dans 50% des cas, à type d'hyperexcitabilité atriale, hyperexcitabilité ventriculaire et dysfonction sinusale (Tableau 2). Aucun cas de BAV paroxystique n'a été noté. Tous nos patients ont eu une ETTqui était normale chez 17 patients avec notamment un VG non dilaté (DTD moyen à 46,5 mm) et une fonction systolo-diastolique correcte (FEVG moyenne à 62%). Le patient P16 présente un prolapsus de la petite valve mitrale responsable d'une insuffisance mitrale grade II. L'ECG haute amplification a été réalisé chez deux patients symptomatiques (P5 et P6) chez qui l'ECG de repos n'objectivait pas de trouble conductif. Ce dernier était normal.

**Tableau 1:** Fréquence des signes cardiaques des patients

Signes fonctionnels cardiovasculaires	Nombre de cas	Pourcentage
Dyspnée	6	33%
Palpitations	4	22%
Syncope	1	5,5%
Lipothymie	5	27,5%
Douleur thoracique	2	11%

**Tableau 2:** Données électrocardiographiques des patients

	Anomalies électriques Pourcentage	Nombre de cas
**ECG de base**	BAV 1^er^ degré 11%	2
	PR à 0,20 s 5,5%	1
	Onde P microvoltée 11%	2
	BBDI 11%	2
	BBGC + BAV 1^er^ degré 5,5%	1
	WPW 5,5%	1
	Onde T négative (V1, V2) 5,5%	1
	Bradycardie à 40cpm 5,5%	1
	Total 61%	11/18
**Holter ECG**	Hyperexcitabilité atriale sans TSV 5,5%	1
	Hyperexcitabilité atriale + ESV stade II LOWN 5,5%	1
	Fibrillation auriculaire 5,5%	1
	Dysfonction sinusale 22%	4
	ESV stade II de LOWN 5,5%	1
	Bradycardie nocturne <40 cpm 5,5%	1
	Total 50%	9/18

BAV: bloc auriculoventriculaire; BBDI: bloc de branche droit incomplet; BBGC: bloc de branche gauche complet; ESV: extrasystoles ventriculaire; TSV: tachycardie supraventriculaire; WPW: syndrome de Wolf Parkinson White

**Tableau 3:** Corrélation entre symptomatologie clinique et signes électriques

Corrélation entre: - Symptômes (lipothymies, syncope, palpitations) et - Trouble du rythme- Trouble de la conduction	Symptômes	Anomalies ECG de base	Anomalies Holter ECG
Cas 3	Asymptomatique	BBDI	Normal
Cas 4	Asymptomatique	WPW	Hyperexcitabilité atriale + ESV stade II de LOWN
Cas 5	Palpitations	Onde T (-) en V1, V2	ESV stade II de LOWN
Cas 6	Lipothymies	Normal	ESSV, BS
Cas 8	Palpitations + Syncope	Onde P microvoltée	Dysfonction sinusale
Cas 9	Lipothymies	BBDI	Dysfonction sinusale
Cas 10 même famille	Asymptomatique	Bradycardie à 40cpm	Dysfonction sinusale
Cas 11	Asymptomatique	Normal	Dysfonction sinusale
Cas 12	Palpitations	BAV 1^er^ degré + BBGC	Non fait (perdu de vue)
Cas 14	Lipothymies	BAV 1^er^ degré	FA paroxystique
Cas 15	Lipothymies	Onde P microvoltée	Normal
Cas 16	Palpitations	PR = 0,20s	Bradycardie sinusale
Cas 17	Lipothymies	BAV 1^er^ degré	Normal

BAV: bloc auriculoventriculaire; BBDI: bloc de branche droit incomplet, FA: fibrillation auriculaire, ESV: extrasystoles ventriculaire, ESSV: extrasystole supraventriculaire, BS: bradycardie sinusale, WPW: syndrome de Wolf Parkinson White

3 patients (P3, P5, P8) ont bénéficiés d'une EEP au début du suivi: P3: patiente asymptomatique et porteuse d'un BBDI. L'EEP est normale; P8: patiente présentant des syncopes et une onde P micro-voltée. L'EEP a objectivé un bloc tronculaire ayant conduit à l'implantation d'un PM DDDR; P5: présente des ondes T négatives en V1 et V2 et une hyperexcitabilité ventriculaire stade II de LOWN. L’épreuve d'effort ainsi que l'ECG haute amplification sont normaux. L'EEP n'a pas objectivé de trouble conductif, avec notamment un temps His-Ventricule à 53 ms, et la patiente a été mise sous BB. A noter que le patient P12 porteur d'un BBGC a été perdu de vue sans pouvoir être exploré. De plus, les patients P9, P10 et P11, présentant une dysfonction sinusale, ainsi que les patients P6 et P16, présentant une bradycardie sinusale paroxystique, n'ont pu bénéficier d'une EPP pour causes sociales.

### Autres atteintes associées

Neufs patients (soit 50% des cas) présentent une cataracte sous-capsulaire postérieure; quatre présentent une dysthyroïdie (3 cas d'hypothyroïdie et un cas d'hyperthyroïdie); quatre patients sont diabétiques (dont un présente également une impuissance sexuelle), deux patients de sexe masculin ont une calvitie précoce et enfin un patient est porteur d'une lithiase de la vésicule biliaire.

### Evolution

Le patient P14 porteur d'un BAV 1^er^ degré et d'une FA paroxystique, a présenté des lipothymies après une année d’évolution et est décédé d'une affection respiratoire avant qu'une EEP n'ait pu être réalisée (manque de moyen). La patiente P8 (porteuse d'un PM DDD) a présenté une syncope après 4 ans d’évolution. Une nouvelle EEP a objectivé un rythme idio-ventriculaire accéléré (RIVA) et elle a été mise sous Sotalol avec une bonne évolution. A noter que sa sœur (connue porteuse de la DM1) est décédée d'une mort subite et a toujours refusé de subir un examen cardiovasculaire de dépistage. Le patient P4 (WPW): accuse des lipothymies et des palpitations après un an d’évolution, avec un BBG sur l'ECG de base. L'ETT a été effectuée montrant une dilatation du VG et une dysfonction systolique modérée à 48% (initialement 56%) avec hypokinésie inférieure et inféro-septale. L'Holter ECG a révélé des ESV stade IV de LOWN. La coronarographie est normale et l'EEP n'a pas objectivé d'anomalies sur les voies de conduction, justifiant l'administration d'un bétabloquant associé à l'IEC.

## Discussion

66% de nos patients présentent des signes fonctionnels cardiaques. Ce taux concorde avec la série de Nishioka portant sur 83 patients dont 56% sont symptomatiques après 6 ans d’évolution [3]. Ce taux peut atteindre 89% à des stades avancés de la maladie [4] et l'atteinte cardiaque peut même être inaugurale dans certains cas. Contrairement aux autres dystrophies musculaires où l’évolution vers une cardiomyopathie dilatée en dysfonction systolique sévère est quasiment inéluctable; dans la maladie de Steinert la fraction d’éjection du ventricule gauche reste longtemps conservée [5] etle pronostic de la DM1 est avant tout lié aux troubles de conduction et tachyarythmies, expliquant un taux de mortalité à peu près 7 fois plus important que dans la population générale [6]. Les autres causes de mortalité seraient un infarctus du myocarde, une embolie pulmonaire et des complications respiratoires (cas du patient 14). Les troubles de conduction sur l'ECG de repos sont les plus fréquemment rencontrés dans notre série avec 16 p.100 de BAV 1er degré et 16 p.100 de trouble de la conduction intraventriculaire. Ces résultats correspondent globalement à ceux de la littérature où le BAV 1er degré est le trouble le plus fréquent (64% dans la série de Chebel [7], 29% dans la série de Nishioka [3]), suivi de trouble de la conduction intra-ventriculaire à type d'hémiblocs, BBD et BBG (32% dans la série de Chebel, 25% dans celle de Nishioka). Ces troubles de la conduction sont le plus souvent en rapport avec des anomalies de la conduction infrahissienne [PM] et plusieurs études ont montré qu'un patient sur deux avait un allongement de l'intervalle HV > 70 ms. La dysfonction sinusale, le plus souvent exprimée sous forme de bradycardie sinusale, est retrouvée dans 22% des cas dans notre série, alors qu'elle est moins fréquente dans les études (1 à 2%) [8, 9].

Les troubles du rythme et de la repolarisation sont plus rares (3 à 21%) [10]: nous avons noté dans notre série un seul cas de trouble de la repolarisation et aucun cas de trouble du rythme sur l'ECG de repos. L'Holter ECG est par contre plus sensible pour la détection des hyperexcitabilités atriales et ventriculaires: 16,5% dans notre série, 26% dans la série d'Antonini [11] et 13% dans celle de Chebel [7]. Par ailleurs, l'Holer ECG permet de dépister un BAV paroxystiquedans 3% des cas [7, 12], une tachycardie ventriculaire dans 3% des cas [13], une fibrillation atriale dans 6 à 9% des cas (5,5% dans notre série) ou encore une dysfonction sinusale (22% de nos patients), passés inaperçus sur le tracé électrique de base. Un ECG haute amplification pourrait également orienter vers la présence d'un trouble conductif puisque son anomalie (QRSD supérieur ou égal à 100 ms et LAS supérieur à 36) est le reflet d'un intervalle HV supérieur à 70 ms avec une bonne sensibilité et spécificité [14]. L'ETT à mis en évidence un prolapsus de la valve mitrale dans un cas (soit 5,5%). Cette anomalie est rapportée dans la littérature chez 0,33 à 33% des patients atteints de DM1 [15]. En outre, la fonction systolique, souvent normale selon la plupart des études [7], n'a été abaissée que chez un seul patient (dont la coronarographie était par ailleurs normale), probablement en rapport avec une baisse de la réserve coronaire et anomalie de la microcirculation. Quant à l'exploration électrophysiologique, ses indications sont larges dans la maladie de Steinert (Tableau 4) [16]. Elle est indiquée principalement en cas de symptômes ou d'anomalies à l'ECG de surface. Cependant, certains auteurs l'ayant pratiquée systématiquement chez tous leurs patients atteints de DM1, ont obtenu un intervalle HV supérieur à 70 ms chez des patients asymptomatiques et avec ECG de surface normal dans 13 à 30% des cas [12, 17]. Dans notre contexte, vus le coût et le caractère invasif de l'exploration du faisceau de His, on réserve cette dernière aux patients répondant aux critères résumées dans le Tableau 4.

**Tableau 4:** Indications de l'EEP

histoire familiale	Mort subite, FV, TV soutenue, PM ou DAI
Symptômes	Palpitations, syncope, vertiges
Anomalies de la conduction sur l'ECG/ Holter ECG/ PVT	BAV 1^er^, 2^ème^ ou 3^ème^ degré, QRS > 120 ms BBG, BBD, hémiblocs PVT positifs
Dysfonction sinusale	Pause sinusale > 3 secondes Bradycardie sinusale < 40/min
Arythmies supra ventriculaires	Tachycardie atriale ACFA/ Flutter
Arythmies ventriculaires	Nombreuses ESV TV soutenue ou non soutenue

Au terme de la description des troubles conductifs, les recommandations de l'ACC et de l'AHA [18] indiquent l'implantation d'un pacemaker en cas de BAV de haut degré ou complet, symptomatique ou non, en cas de déficience sinusale avec une pause supérieure à trois secondes et en cas de bloc bifasciculaire avec intervalle HV supérieur à 100 ms (Classe I). D'autres indications sont fortement recommandées: patient se plaignant de syncope avec un intervalle HV supérieur ou égal à 100 ms (Classe II a) ou les patients ayant des arythmies atriales symptomatiques avec un intervalle HV long et qui nécessitent un traitement antiarythmique. Certains auteurs plaident pour l'implantation d'un pacemaker prophylactique en cas de découverte à l'EEP d'un intervalle HV supérieur à 70 ms chez un patient par ailleurs asymptomatique [7, 9]. Cependant, le pacemaker n’épargne pas une mort subite d'origine rythmique ventriculaire et il n'existe pas de recommandations à ce jour quant à l'implantation d'un défibrillateur automatique implantable en cas de DM1. Ce dernier est indiqué en prévention secondaire en cas d'arrêt cardiaque sur TV ou FV, ou chez les patients relevant de l'implantation d'un pacemaker et qui présentent une dysfonction ventriculaire gauche ou une tachycardie ventriculaire documentée. L’évolution globale de la maladie de Steinert se fait vers un état grabataire à un âge plus tardif par rapport aux autres dystrophies musculaires, après une durée moyenne d’évolution de 28,4 ans [19]. Aucun de nos patients n'est grabataire, même après 25 ans d’évolution.

## Conclusion

L'atteinte cardiaque au cours de la maladie de Steinert est fréquente, évolutive, non corrélée à l'atteinte neuro-musculaire, et souvent responsable d'une mort subite de cause rythmique. Tout cardiologue doit être averti des complications cardiaques et soumettre son patient à un bilan cardiovasculaire minutieux, annuel ou bi-annuel, afin de dépister les troubles rythmiques et/ou conductifs et d'instaurer un traitement à temps. Inversement, devant tout trouble du rythme ou de la conduction du sujet jeune, un examen neurologique s'impose à la recherche d'une dystrophie musculaire type Steinert.
